# Music-Inspired MK545Pat for AF/PAF/Non-AF Classification Using ECG Signals

**DOI:** 10.3390/bioengineering13091016

**Published:** 2026-08-31

**Authors:** Irem Ece Gulensoy, Ilknur Tuncer, Tarik Kivrak, Omer Faruk Goktas, Mehmet Baygin, Prabal Datta Barua, Sengul Dogan, Mehmet Ali Kobat, Turker Tuncer

**Affiliations:** 1 Department of Music, Faculty of Fine Arts, Kocaeli University, 41001 Kocaeli, Türkiye; irem.gulensoy@kocaeli.edu.tr; 2 Software and Informatics Research Institute, Firat University, 23119 Elazig, Türkiye; ituncer@firat.edu.tr; 3 Department of Cardiology, Firat University Hospital, Firat University, 23119 Elazig, Türkiye; mkobat@firat.edu.tr; 4 Department of Electronics and Automation, Technical Sciences Vocational School, Ankara Yildirim Beyazit University, 06760 Ankara, Türkiye; ofgoktas@aybu.edu.tr; 5 Department of Computer Engineering, Faculty of Engineering and Architecture, Erzurum Technical University, 25050 Erzurum, Türkiye; mehmet.baygin@erzurum.edu.tr; 6 School of Business (Information System), University of Southern Queensland, Toowoomba, QLD 4350, Australia; prabal.barua@usq.edu.au; 7 Department of Digital Forensics Engineering, Technology Faculty, Firat University, 23119 Elazig, Türkiye; sdogan@firat.edu.tr (S.D.); turkertuncer@firat.edu.tr (T.T.)

**Keywords:** music-inspired pattern, atrial fibrillation, paroxysmal atrial fibrillation, ECG, deterministic feature engineering, ablation, segment-level evaluation, patient-wise LOSO

## Abstract

Paroxysmal atrial fibrillation (PAF) may be missed on intermittent electrocardiographic (ECG) recordings. We evaluated MK545Pat, a deterministic one-dimensional descriptor derived from the first-appearance order of the notes in Mozart’s Piano Sonata K.545. The CPSC 2021 dataset contains 1436 records from 105 patients, from which 116,042 15 s segments were analyzed. Each segment produced 2304 histogram features from 2942 unit-stride 59-sample blocks. Segment-level 10-fold cross-validation achieved 97.49 ± 0.18% accuracy (range, 97.16–97.71%), and a separate 90:10 segment-level holdout achieved 97.49% accuracy and 95.85% macro-F1. A separate patient-wise leave-one-subject-out (LOSO) analysis held out all records and segments from one patient at a time. The unweighted mean of the 105 held-out patient-specific segment accuracies was 71.29% (median, 78.17%; SD, 24.22%; range, 0.00–96.00%). Pattern controls were lower for identity, reverse, and interleaved assignments, while a within-group permutation retained 97.49%, as predicted by the invariance analysis. The results support MK545Pat as a reproducible feature-engineering approach and show the expected performance gap between segment-level and cross-patient evaluation.

## 1. Introduction

Atrial fibrillation (AF) is the most common sustained arrhythmia encountered in clinical practice [1]. It is characterized by rapid, disorganized atrial depolarization and an irregular ventricular response [2]. Loss of coordinated atrial contraction promotes blood stasis and thrombus formation, while inflammatory and hemostatic mechanisms may further contribute to thrombogenic risk [3]. Oral anticoagulation reduces thromboembolic events in appropriately selected patients [4], but treatment must be balanced against bleeding risk [5]. Automated ECG analysis has also been investigated with neural network and random forest approaches [6,7]. Clinical diagnosis should be confirmed on ECG before treatment decisions are made [4].

AF is commonly described as paroxysmal, persistent, long-standing persistent, or permanent, using episode duration and rhythm management strategy [4,8]. PAF terminates spontaneously or with intervention within 7 days. Episodes may be brief, intermittent, and asymptomatic, making them difficult to capture on intermittent ECG recordings [9,10]. AF burden also matters: greater burden and progression are associated with worse outcomes [11,12], and no single-episode duration threshold applies uniformly across patients [13]. Anticoagulation decisions should therefore follow clinical thromboembolic-risk assessment rather than episode duration alone [4,14,15].

AF is identified on ECG by characteristic atrial activity and an irregular ventricular response; fibrillatory-wave and RR-based features have both been studied [16,17]. Noise and other irregular rhythms can obscure these cues. ECG-based AF analysis has also been reviewed as a computer-aided diagnosis problem [18]. During non-AF rhythm, P-wave morphology has been explored as a marker of PAF risk [19,20], and HRV-based methods have been used for PAF prediction [21,22,23]. These signals may support risk assessment, but they do not replace ECG confirmation of AF.

In cryptogenic stroke, longer ECG monitoring increases AF detection yield [24] but also increases the signal review burden. Automated analysis can help manage this workload. We evaluate MK545Pat as a deterministic direct-waveform descriptor for three-class AF/PAF/non-AF classification on CPSC 2021 [25,26].

### 1.1. Related Works

Table 1 places MK545Pat beside related ECG methods, although the tasks and validation units differ. Teijeiro et al. addressed record- and heartbeat-level arrhythmia classification [27,28]; Peimankar and Puthusserypady developed DENS-ECG for ECG delineation [29]; and Sorayaie Azar et al. used a spectrogram vision transformer for short-term AF detection [30]. Additional studies provide context from P-wave detection [31], Bayesian AF detection [32], RR interval deep learning [33], AF detection reviews [34], and group-aware short-term classification [35]. These studies are relevant, but their headline metrics are not direct benchmarks for the present three-class segment-level task.

### 1.2. Motivation and Theoretical Rationale

MK545Pat was designed to be deterministic and easy to inspect. The musical score is used only as a fixed indexing source; no physiological link between Mozart’s music and cardiac electrophysiology is assumed.

ECG is an ordered waveform. Local rises, falls, repetitions, and abrupt transitions can be encoded by comparing nearby samples. MK545Pat makes these comparisons within 59-sample blocks and collects the resulting binary patterns in histograms.

K.545 is not assumed to be physiologically special. Its first-movement score simply provides a fixed sequence that can be reproduced exactly. The control-pattern ablation tests whether the resulting position partition matters.

After unused note symbols are removed, K.545 yields a 58-index permutation. These indices assign the 58 non-pivot samples of each 59-sample block to seven-bit groups of sizes 8, 8, 8, 8, 8, 9, and 9. The relevant computational choice is the assignment of sample positions to groups. Reordering positions within one group only relabels histogram bins; moving positions across groups changes the partition.

Within each block, MK545Pat reorders 58 non-pivot samples, compares each with the pivot, and maps the binary responses to seven histograms. It is therefore a fixed local comparison operator rather than a learned representation.

MK545Pat works directly on waveform samples and does not require R-peak detection or HRV calculation. Its fixed output and explicit histogram groups make the representation easy to inspect.

The musical score provides a reproducible index structure. The experiments test whether this fixed partition is useful for ECG discrimination at both segment and patient-wise validation levels.

Segment-level performance was evaluated with 10-fold cross-validation and a separate 90:10 holdout. Cross-patient performance was evaluated separately with patient-wise LOSO.

The validation units, aggregation rule, and evaluation metrics are detailed in Section 2.2.2.

### 1.3. Contributions

This study makes four contributions:

• We define a deterministic 58-index music-derived operator with seven grouped histograms and prove its within-group permutation invariance.
• MK545Pat produces a 2304-feature direct-waveform vector for each 15 s ECG segment without R-peak detection or HRV computation.
• The complete segment-level pipeline reached 97.49 ± 0.18% accuracy across 10 folds, while the separate patient-wise LOSO analysis yielded 71.29% as the equally weighted mean of 105 held-out patient-specific segment accuracies. Histogram, pattern, and classifier ablations examined the main pipeline components.
• The ablations separate histogram, position partition, and classifier effects; the analytical invariance also identifies an information-equivalent control.

## 2. Materials and Methods

### 2.1. Materials

The CPSC 2021 ECG dataset was obtained from PhysioNet [25,26]. The challenge files provide lead I and lead II signals sampled at 200 Hz. We used the supplied ECG signals directly, without lead reconstruction or fusion. No additional signal-level filtering, denoising, amplitude normalization, or resampling was applied. Each signal was divided into consecutive non-overlapping 3000-sample windows (15 s; stride, 3000 samples). The dataset contains 1436 variable-length records from 105 patients. Stage I contains 730 records from 54 patients: 7 persistent-AF, 5 PAF, and 42 non-AF. Stage II contains 706 records from 51 patients: 19 persistent-AF, 18 PAF, and 14 non-AF. The combined set therefore contains 26 patients with persistent-AF, 23 with PAF, and 56 with non-AF. The segmentation produced 116,042 15-s samples: 35,181 AF, 10,918 PAF, and 69,943 non-AF. Each segment inherits its record label. A PAF-labeled segment is not assumed to contain an AF episode; the task is record-label-derived AF/PAF/non-AF classification, not AF onset or end localization. Dataset details are summarized in Table 2.

Three validation settings were used. The stratified 10-fold analysis and the separate 90:10 holdout were segment-level evaluations. The holdout contained 11,604 segments: 6994 non-AF, 3518 AF, and 1092 PAF, and it was not one of the 10 cross-validation folds. Patient-wise LOSO was evaluated separately. One patient defined each of 105 test folds; all records and all 15 s segments from that patient were excluded from training, and the remaining 104 patients formed the training set.

### 2.2. Methods

#### 2.2.1. MK545Pat Pattern Construction

To build the index pattern, we read the melodic line of the first movement of Mozart’s Piano Sonata K.545 in temporal order. Note duration was ignored. Notes were mapped to an ordered list of natural, sharp, and flat pitches across the treble and bass clefs. Unused symbols were removed, and the remaining notes were renumbered. The resulting first-appearance sequence defines MK545Pat. The construction is given below.

**Figure 1:**
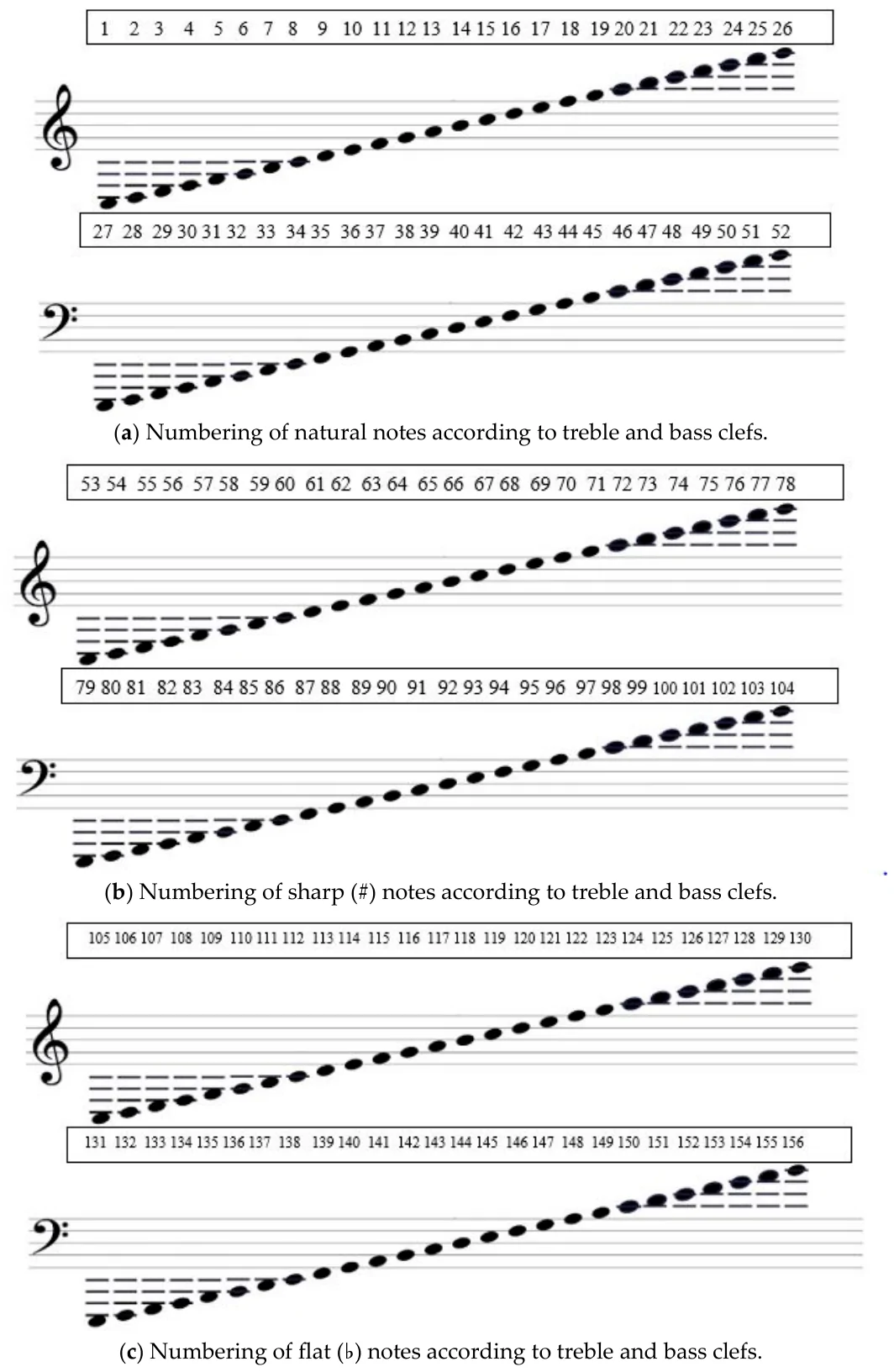
Enumeration list of the notes.

Step 1: Enumerate notes of MK545 according to Figure 1.

Step 2: Eliminate unused notes.

**Figure 2:**
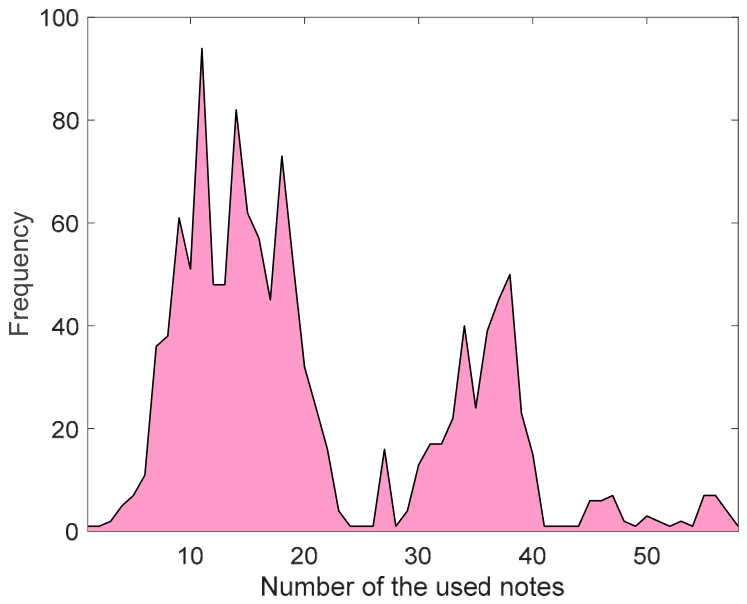
Frequency histogram of 58 notes retained from K.545.

Step 3: Renumber the used notes. The histogram of the used notes is depicted in Figure 2.

Step 4: Build the MK545Pat index from the enumerated melody. Algorithm 1 scans the note sequence once and stores each used note index at its first appearance. The process is deterministic: it uses no random generator, seed, or substitution-box sampling. Table 3 lists the resulting 58-value permutation.
**Algorithm 1.** Pattern generator.**Input:** The enumerated note list. **Output:** The created pattern1: $control=zeros\left(58\right);$ 2: i=0; j=0; 3: $tpl=\sum_{t=1}^{58}control\left(t\right);$ 4: **while** tpl<58 **do** 5: $value=notes\left(i\right);$ 6: **if** $control\left(value\right)=0$ **then** 7:  $control\left(value\right)=1;$ 8:  $pattern\left(j\right)=value$; 9:  j=j+1; 10: **end if**
11: $tpl=\sum_{t=1}^{58}control\left(t\right);$
12: i=i+1; 13: **end while**

Table 3 lists the resulting MK545Pat pattern.

Step 1: Apply a unit-stride overlapping block division with length 59 within each ECG segment. For block i, positions j = 1, …, 59 follow a single 1-based convention. The 59th element is the pivot, and positions 1–58 are the only samples addressed by the MK545Pat permutation.

${bl}_{i}$ is the i-th 59-sample block, s is the ECG segment of length L, and ${piv}_{i}$ is the pivot sample.

Step 2: Generate 58 binary comparisons per block using the deterministic MK545Pat permutation and the pivot, as defined in Equations (3) and (4).

(1) $${bl}_{i}\left(j\right)=s\left(i+j-1\right),\;i\in \{1,\ldots ,L-58\},\;j\in \{1,\ldots ,59\}$$

(2) $${piv}_{i}={bl}_{i}\left(59\right)$$

where ${bit}_{i}\left(r\right)$ is the r-th binary comparison, and $pattern\left(r\right)$ selects one non-pivot sample position.

Step 3: Divide the 58 binary comparisons into seven fixed groups with sizes (8, 8, 8, 8, 8, 9, 9), as defined in Equations (5) and (6), where $G_{1},\ldots ,G_{7}$ are the seven fixed bit groups. The first five groups contain eight bits and the last two groups contain nine bits.

(3) $${bit}_{i}\left(r\right)=Signum\left({bl}_{i}\left(pattern\left(r\right)\right),{piv}_{i}\right),\;r\in \{1,\ldots ,58\}$$

(4) $$Signum\left(x,p\right)=\left\{\begin{array}{ll} 0, & x<p\\ 1, & x\geq p \end{array}\right.$$

For any permutation π satisfying $\pi \left(G_{g}\right)=G_{g}$ for every group g, the descriptor satisfies $H_{\pi }\left(s\right)=P_{\pi }H\left(s\right)$, where $P_{\pi }$ only permutes histogram bins. Thus, within-group permutations are information-equivalent controls; cross-group permutations change the position partition.

Step 4: Calculate map signals using these groups.

(5) $$G_{k}={\left\{{bit}_{i}\left(8\left(k-1\right)+h\right)\right\}}_{h=1}^{8},\;k=1,\ldots ,5$$

(6) $$G_{5+t}={\left\{{bit}_{i}\left(40+9\left(t-1\right)+g\right)\right\}}_{g=1}^{9},\;t=1,2$$

(7) $$m_{i}^{\left(k\right)}=\sum_{r=1}^{8}G_{k}\left(r\right)2^{r-1},\;k=1,\ldots ,5$$

(8) $$m_{i}^{\left(k\right)}=\sum_{r=1}^{9}G_{k}\left(r\right)2^{r-1},\;k=6,7$$

where $m_{i}^{\left(k\right)}$ denotes the group-wise map signal.

Step 5: Extract histograms from map signals.

Step 6: Concatenate the seven histograms. The five 8-bit maps contribute 5 × 256 bins, and the two 9-bit maps contribute 2 × 512 bins, giving 2304 features in total. Equations (1)–(13) define the complete descriptor.

#### 2.2.2. MK545Pat Classification Pipeline

**Figure 3:**
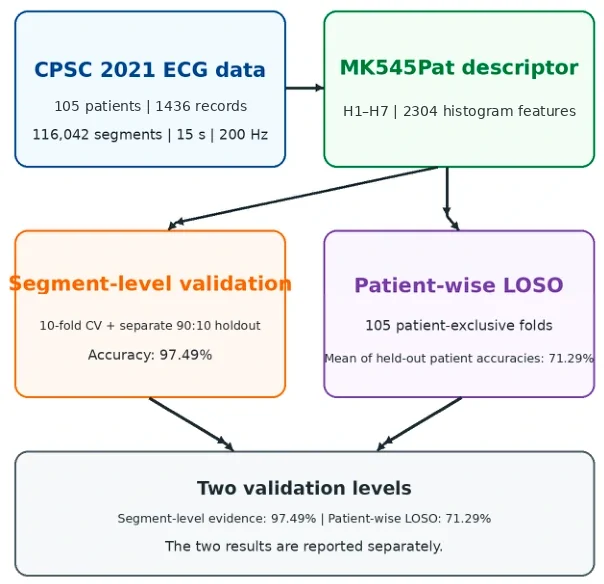
MK545Pat feature selection and classification workflow.

The same MK545Pat-NCA-SVM pipeline was used for the reported evaluations (Figure 3). MK545Pat extraction is deterministic. Within each training partition, neighborhood components analysis (NCA) [46] ranked the 2304 features, and the top 1000 training-ranked features were classified with a degree-3 polynomial-kernel support vector machine (SVM) [47]. For patient-wise LOSO, all records and segments from the held-out patient remained outside standardization, NCA fitting, feature selection, and SVM training.

Step 1: Generate the 2304-dimensional MK545Pat vector for each ECG segment, as shown in Equation (9).

(9) $$f_{n,j}=MK545Pat{\left(s_{n}\right)}_{j},\;n=1,\ldots ,N,\;j=1,\ldots ,2304$$

Step 2: For each split, estimate the feature-standardization parameters from the training data and apply them unchanged to the test data. Fit NCA on the standardized training data, and use the resulting ranking for feature selection. Equations (10)–(12) define this procedure.

(10) $$S_{train}^{\left(u\right)}\cap S_{test}^{\left(u\right)}=⌀,\;u=1,\ldots ,105$$

(11) $$z_{ij}=\frac{x_{ij}-\mu_{j}^{\left(train\right)}}{\sigma_{j}^{\left(train\right)}}$$

(12) $$idx=NCA\left(Z_{train},y_{train}\right)$$

Here, $x_{ij}$ and $z_{ij}$ denote the original and standardized value of feature j for training sample i; $\mu_{j}^{\left(train\right)}$ and $\sigma_{j}^{\left(train\right)}$ are estimated from the training fold only; idx is the NCA ranking; k* = 1000.

Step 3: Retain the first 1000 NCA-ranked features from the training fold. Apply the same indices to the test fold, as stated in Equation (13).

We report accuracy, macro precision, macro recall, macro-F1, and class-wise recall, precision, and F1 for the segment-level analyses. No resampling or class weighting was used. For patient-wise LOSO, segment accuracy was calculated separately for each held-out patient, and the 105 patient-specific accuracies were averaged with equal patient weight. Thus, 71.29% is a patient-exclusive LOSO result at the segment prediction unit; it is not one label per patient majority-vote accuracy.

(13) $$f_{n,z}^{*}=f_{n,idx\left(z\right)},\;z=1,\ldots ,k^{*}$$

Step 4: Fit the degree-3 polynomial-kernel SVM and evaluate the test partition. In the LOSO analysis, one patient is the complete test set for each fold. All predictions for that patient are generated by a model fitted only on the other 104 patients. For the segment-level 10-fold analysis, the mean, sample SD, and range of fold accuracies are reported.

The CPSC 2021 signals were used at their native 200 Hz sampling rate. No signal-level filtering, denoising, amplitude normalization, or resampling was applied before MK545Pat. The 15 s windows were non-overlapping 3000-sample segments. The unit-stride 59-sample operation described in Section 2.2.1 is internal to the MK545Pat descriptor and is separate from the 15 s segmentation stride. Supplementary File S1 provides the full MATLAB (2023b) implementation and the patient-wise LOSO code.

## 3. Results

### 3.1. Segment-Level Classification

**Figure 4:**
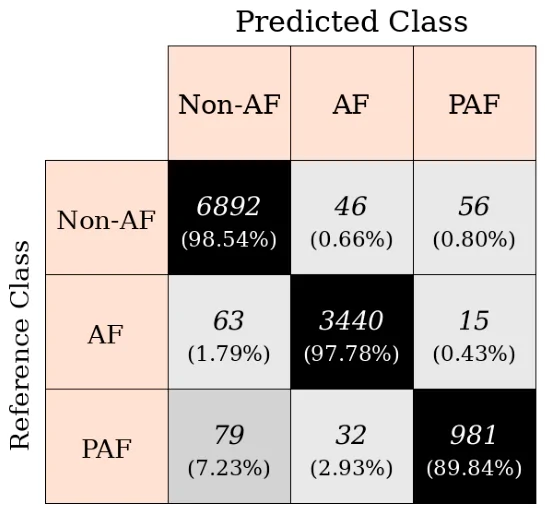
Segment-level holdout confusion matrix. Rows are reference classes, and columns are predicted classes. PAF recall was 89.84%.

The separate 90:10 segment-level holdout contained 11,604 segments: 6994 non-AF, 3518 AF, and 1092 PAF. The confusion matrix included 11,313 correct decisions, giving 97.49% accuracy (Figure 4). Macro precision, macro recall, and macro-F1 were 96.34%, 95.39%, and 95.85%, respectively (Table 4).

The non-AF class accounted for 6994 of the 11,604 holdout segments (60.27%), providing a simple reference for the class imbalance.

**Figure 5:**
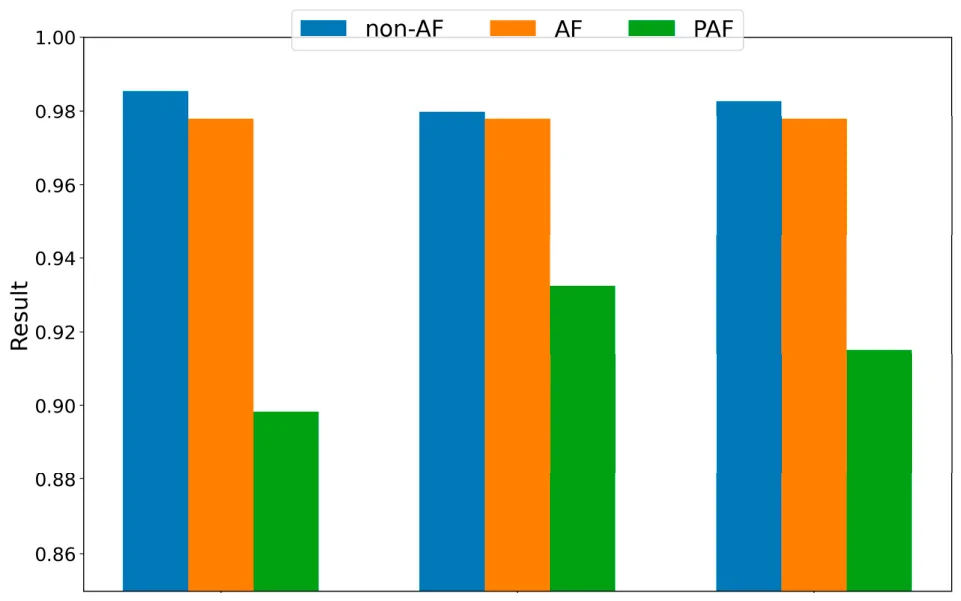
Segment-level recall, precision, and F1 by class.

Non-AF, AF, and PAF recall values were 98.54%, 97.78%, and 89.84%. Their precision values were 97.98%, 97.78%, and 93.25%, and their F1 values were 98.26%, 97.78%, and 91.51%, respectively (Figure 5). PAF was the weakest class, consistent with its smaller segment count.

### 3.2. Fold Stability

The complete H1–H7 model reached 97.49 ± 0.18% accuracy across the 10 segment-level folds, with fold accuracies ranging from 97.16% to 97.71% (Table 5).

The non-AF class accounted for 69,943 of the 116,042 segments (60.27%). Every segment-level fold accuracy exceeded this majority-class reference.

### 3.3. Histogram Ablation

H6 was the strongest single histogram (90.08%) and H2 the weakest (83.02%) (Table 6). The full H1–H7 descriptor was 7.41 points higher than H6.

Accuracy increased from 83.68% with H1 to 97.49% with H1–H7 (Table 7). The largest gain was 7.34 percentage points after H2 was added.

### 3.4. Pattern and Classifier Ablation

At the segment level, MK545Pat exceeded the identity, reverse, and interleaved patterns by 4.82, 4.54, and 1.93 percentage points, respectively (Table 8). The within-group permutation retained 97.49%, matching the histogram-bin relabeling invariance in Equations (5)–(8).

The full NCA-cubic-SVM pipeline achieved 97.49% (Table 9). Cityblock kNN reached 95.23% and linear SVM 93.12%; tree ensembles and Gaussian naïve Bayes were lower. The best result therefore depends on both descriptor and classifier choice.

Table 6, Table 7, Table 8 and Table 9 summarize the behavior of the descriptor components and classifier choices under the same segment-level evaluation setting.

### 3.5. Patient-Wise LOSO Validation

**Figure 6:**
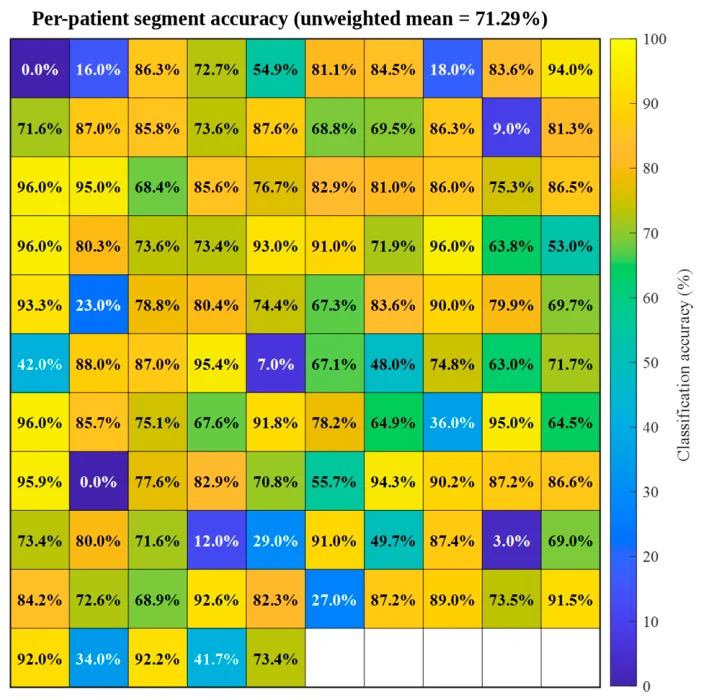
Patient-specific held-out segment accuracy across 105 LOSO folds. Each filled cell represents one held-out patient; five empty cells are layout padding. The equally weighted mean was 71.29%, with a median of 78.17%, an SD of 24.22%, and a range of 0.00–96.00%. Each patient contributed equally to the reported LOSO summary.

Patient-wise LOSO yielded 71.29% as the equally weighted mean of the 105 held-out patient-specific segment accuracies. The median was 78.17%, the SD was 24.22%, and the range was 0.00–96.00% (Figure 6). In every fold, all records and segments from one patient were excluded from model fitting and used only for testing.

## 4. Discussion

Segment-level performance was consistent across the tested partitions. The 10-fold mean matched the separate holdout accuracy, and fold variation was small. PAF remained the hardest class, with 89.84% recall and 91.51% F1 in the holdout.

The patient-wise LOSO result gives a different and more demanding view of generalization. Mean held-out patient-specific segment accuracy was 71.29%, 26.20 percentage points below the segment-level of 97.49%. This gap shows why the segment-level and patient-wise results should be interpreted separately. The segment-level class-wise results also reflect the class imbalance, with PAF showing the lowest recall and F1.

The histogram ablation indicates complementary information across H1–H7. H6 was the strongest single component, but the complete descriptor was 7.41 percentage points higher. Accuracy also increased at each cumulative stage.

The pattern controls agreed with the invariance analysis. Cross-group changes altered segment-level accuracy, while a within-group permutation preserved it exactly. This supports the interpretation of the musical sequence as a fixed indexing rule rather than a physiological mechanism.

Classifier choice also affected performance. NCA with cubic SVM was the strongest tested configuration, while the same descriptor did not performed as well as the alternatives in Table 9. The final performance therefore reflects both the descriptor and the classifier.

The studies in Table 1 provide useful context but address different endpoints, datasets, and validation units [31,32,33,34,35]. Their reported scores should therefore be interpreted as literature context rather than direct comparisons.

### 4.1. Strengths and Practical Advantages

MK545Pat has five practical advantages: deterministic extraction, direct waveform input, a fixed 2304-feature output, no R-peak or HRV prerequisite, and explicit histogram groups for component-level analysis.

The fixed partition can be regenerated exactly and supports an analytical invariance test. Feature extraction, NCA ranking, and SVM classification are separate stages, so each stage can be inspected or replaced independently.

### 4.2. Limitations and Future Work

Several limitations remain. First, the retained patient-wise LOSO evidence includes the 71.29% summary and the distribution of the 105 held-out patient-specific accuracies, but the original fold-level class scores, LOSO confusion matrix, class-wise LOSO metrics, and confidence intervals were not retained. Second, the 97.49% analyses are segment-level and may retain patient-specific information; they should not be interpreted as cross-patient performance. The separate 71.29% LOSO result is the appropriate patient-wise estimate in this study.

Third, the CPSC 2021 lead I and lead II signals were used directly at 200 Hz, without additional signal-level filtering, denoising, amplitude normalization, or resampling, and were divided into non-overlapping 3000-sample windows. Fourth, PAF labels are inherited from the source record and do not indicate AF-positive content in every 15 s segment. Fifth, no external cohort or calibration analysis was performed. Sixth, Table 6, Table 7, Table 8 and Table 9 are segment-level ablations; matched pattern and conventional baselines were not repeated under patient-wise LOSO.

Future studies should retain the full prediction file from every LOSO fold and report class-wise LOSO metrics, a patient-level confusion matrix where a patient-level aggregation rule is prespecified, and patient-level uncertainty intervals. Matched baselines should be rerun on the same LOSO folds. External validation, calibration, runtime, and memory measurements would provide the next level of evidence.

## 5. Conclusions

MK545Pat maps a fixed musical note order to a 2304-feature local ECG descriptor. Segment-level 10-fold accuracy was 97.49 ± 0.18%, and the separate 90:10 holdout reached 97.49% accuracy with 95.85% macro-F1. A separate patient-wise LOSO analysis yielded 71.29% as the equally weighted mean of held-out patient-specific segment accuracies. The lower LOSO value shows greater difficulty of cross-patient generalization. Histogram, pattern, and classifier ablations support the internal behavior of the segment-level pipeline, while the LOSO result defines its current patient-wise performance. MK545Pat is therefore a reproducible feature-engineering method whose clinical generalizability should be tested further in external cohorts.

## Figures and Tables

**Table 1:** Representative automated ECG studies relevant to AF and PAF analysis.

Author(s)	Method	Aim	Dataset	Results (%)	Limitations
Peimankar 2019 [31]	Ensemble LSTM/BiLSTM + Dempster-Shafer	P-wave detection	PhysioNet QT Database (105 recordings)	Acc 98.48 Sen 97.22 PPV 99.94 F1 98.56	P-wave endpoint; not AF/PAF classification
Martis 2013 [32]	DWT + ICA + Bayesian classifiers	AF detection	MIT-BIH Arrhythmia + MIT-BIH AF databases	NB Sen 99.32 Spe 99.33	Binary AF detection; different handcrafted pipeline
Andersen 2019 [33]	CNN + RNN on RR intervals	Real-time AF detection	Three public ECG databases; 89 subjects	CV Sen 98.98 Spe 96.95 Unseen Sen 86.04 Spe 98.96	Binary AF/NSR; RR-interval input
Faust 2020 [34]	Review	AF detection methods as a service	Review of AF detection literature	Not applicable	Review article; no new classifier
Jahan 2022 [35]	SVM, DT, RF, stacking, XGBoost, AdaBoost	Short-term AF detection	23 long ECG records + three unseen datasets	Best Sen 85.67 Spe 81.25 PPV 90.85 F1 88.18	Binary AF/NSR; group-aware validation
Dang 2019 [36]	CNN + BLSTM	AF/ normal	MIT-BIH AF database [37]	Acc 96.59 Sen 99.93 Spe 97.03	AF/non-AF classification only, although dataset contains PAF
Acharya 2017 [38]	CNN	AF/ atrial flutter/ ventricular fibrillation/ normal	MIT-BIH AF database [37], MIT-BIH arrhythmia [39], Creighton University ventricular tachyarrhythmia database	2-s segment: Acc 92.5 Sen 98.09 Spe 93.13 5-s segment: Acc 94.9 Sen 99.13 Spe 81.44	PAF not considered
Ebrahimzadeh 2018 [22]	Linear, non-linear and time-frequency feature extraction, local subset feature selection, MLP, kNN and SVM	PAF prediction	PAF prediction database [40]	Acc 98.2 Sen 100 Spe 95.5	High complexity, HRV signals used
Narin 2018 [23]	HRV time and frequency domain features, feature selection with genetic algorithm, kNN	PAF prediction	PAF prediction database [40]	Acc 90 Sen 92 Spe 88	High complexity, relatively low accuracy
Pourbabaee 2018 [41]	CNN, kNN	PAF screening	Private dataset	Acc 91 Sen 90.2 Pre 90.79	High complexity, dataset not shared
Wang 2021 [42]	Improved quantum particle swarm optimization, SVM	AF/PAF classification	MIT-BIH AF database [37], PAF prediction challenge database [40], custom dataset	MIT-BIH: Acc 99.2 Sen 99.2 Spe 99.2 PAF dataset: Acc 92.5 Sen 93.3 Spe 91.7 Custom dataset: Acc 87.0 Sen 94.2 Spe 79.7	Degraded accuracy for PAF classification
Liaqat 2020 [43]	CNN and classical machine learning algorithm	AF classification	MIT-BIH AF database [37], PhysioNet dataset	Acc 86.5 Pre 86.0 Rec 85.0 F1 86.0	AF classification only, relatively low accuracy
Yue 2021 [44]	Frequency slice wavelet transform, SVM	AF classification	MIT-BIH AF database [37]	Acc 98.15 Sen 96.43 Spe 100	AF classification only
Wei 2019 [45]	Recurrence complex network, CNN, majority voting	AF classification	MIT-BIH AF database [37]	Acc 94.59 Sen 94.28 Spe 94.91	AF classification only
Teijeiro 2017 [27]	Abductive features + RNN stacking	Arrhythmia classification	PhysioNet/CinC 2017	Challenge study	Different task/data; context only
Teijeiro 2018 [28]	Abductive abstract features	Heartbeat classification	MIT-BIH Arrhythmia	See original study	Heartbeat-level task
Peimankar 2021 [29]	CNN-LSTM (DENS-ECG)	ECG delineation	Two public ECG sets	See original study	Not AF/PAF classification
Sorayaie Azar 2025 [30]	Spectrogram + Vision Transformer	Short-term AF detection	Study ECG datasets	CV Acc 98.14; F1 95.81; unseen Acc 95.97; F1 91.14	Different task/data; context only

Acc, accuracy; BLSTM, bidirectional long short-term memory; CNN, convolutional neural network; DT, decision tree; F1, F1 score; HRV, heart rate variability; kNN, k-nearest neighbor; MLP, multilayer perceptron; PPV, positive predictive value; Pre, precision; Rec, recall; RF, random forest; RNN, recurrent neural network; Sen, sensitivity; Spe, specificity; SVM, support vector machine.

**Table 2:** Details of ECG dataset used.

Feature	Value
Number of records	1st stage: 730 records 2nd stage: 706 records
Patient distributions	Stage I: 7 persistent-AF, 5 PAF, 42 non-AF Stage II: 19 persistent-AF, 18 PAF, 14 non-AF
Diagnostic classes	AF; PAF; non-AF
Segment length	15 s
Number of ECG segments	116,042
Signal distribution	35,181 AF; 10,918 PAF; 69,943 non-AF
Combined patient classes	26 persistent-AF; 23 PAF; 56 non-AF; total 105

**Table 3:** The generated 58-value MK545Pat pattern.

14	16	18	13	15	19	21	7	11	9
8	10	12	17	6	37	33	32	46	31
36	30	20	34	35	50	27	29	22	52
38	47	5	45	4	43	56	55	49	23
57	39	53	41	54	42	44	26	28	40
48	51	24	3	1	2	58	25		

**Table 4:** Overall segment-level holdout performance of MK545Pat model.

Performance Metric	Result (%)
Accuracy	97.49
Macro precision	96.34
Macro recall	95.39
Macro-F1	95.85

**Table 5:** Fold-wise segment-level accuracy of complete H1–H7 model.

Fold	Accuracy (%)
1	97.16
2	97.40
3	97.43
4	97.71
5	97.40
6	97.71
7	97.43
8	97.37
9	97.63
10	97.66
Mean ± SD	97.49 ± 0.18

**Table 6:** Single-histogram ablation under segment-level evaluation setting.

Histogram	Accuracy (%)	Difference from Full (Points)
H1	83.68	−13.81
H2	83.02	−14.47
H3	84.01	−13.48
H4	86.66	−10.83
H5	88.54	−8.95
H6	90.08	−7.41
H7	88.65	−8.84
Full H1–H7	97.49	+0.00

**Table 7:** Cumulative histogram ablation under segment-level evaluation setting.

Histograms	Accuracy (%)	Increment (Points)
H1	83.68	-
H1–H2	91.02	+7.34
H1–H3	92.95	+1.93
H1–H4	95.06	+2.11
H1–H5	96.22	+1.16
H1–H6	96.72	+0.50
H1–H7	97.49	+0.77

**Table 8:** Pattern ablation under segment-level evaluation setting.

Pattern	Accuracy (%)	Difference from MK545Pat (Points)
MK545Pat	97.49	+0.00
Identity 1–58	92.67	−4.82
Reverse 58–1	92.95	−4.54
Interleaved	95.56	−1.93
Within-group permutation	97.49	+0.00

**Table 9:** Classifier ablation under segment-level evaluation setting.

Classifier	Accuracy (%)	Difference from Full (Points)
Full model: NCA + cubic SVM	97.49	+0.00
Nearest neighbor, cityblock	95.23	−2.26
Linear SVM	93.12	−4.37
Random forest	89.09	−8.40
Extra trees	88.09	−9.40
Gaussian naïve Bayes	84.40	−13.09

## Data Availability

The CPSC 2021 dataset is publicly available through PhysioNet [25,26] (https://doi.org/10.13026/ksya-qw89). The MK545Pat sequence, segment-level fold accuracies, holdout confusion matrix, ablation results, and patient-wise LOSO summary are reported in the manuscript. Supplementary File S1 provides the MATLAB source code for the descriptor and LOSO evaluation. Derived analysis files and the final implementation code will be released in a public repository after publication.

## Supplementary Materials

The following supporting information can be downloaded at: https://www.mdpi.com/article/10.3390/bioengineering13091016/s1. Supplementary File S1 provides the complete MATLAB source code for MK545Pat feature extraction and the patient-wise LOSO procedure used for the reported 71.29% validation result. It also defines the exported fold-level segment and provides patient summaries.
