# Supplementary material for: Music-Inspired MK545Pat for AF/PAF/Non-AF Classification Using ECG Signals
Source: Bioengineering (Basel). 2026 Aug 31;13(9):1016. doi: 10.3390/bioengineering13091016 (PMC13603543; doi:10.3390/bioengineering13091016)
Supplement: Supplementary file 1 [file bioengineering-13-01016-s001.zip › bioengineering-4444729-supplementary.pdf]

Supplementary File S1

MK545Pat Source Code and Patient-Wise LOSO Reproducibility Details

Scope. This file documents the MK545Pat feature extractor and the patient-wise leave-one-subject-out (LOSO) procedure used for the reported 71.29% result. The code keeps every record and every 15-s segment from the held-out patient outside model fitting. It also exports segment-level and patient-level summaries for audit and reuse.

S1.1. ECG input and data preparation

- Source: CPSC 2021 ECG dataset. The challenge files provide lead I and lead II signals.
- Sampling rate: native 200 Hz.
- Signal-level preprocessing: none. No additional filtering, denoising, amplitude normalization, or resampling was applied before MK545Pat.
- Segmentation: consecutive non-overlapping 15-s windows; 3,000 samples per segment; stride = 3,000 samples.
- The 59-sample unit-stride operation is internal to MK545Pat and is separate from the 15-s segmentation stride.
- LOSO grouping variable: patient identity. All records and all segments from one patient remain in the same held-out fold.

S1.2. Definition of the reported 71.29% LOSO result

The patient-wise LOSO analysis contains 105 outer folds, one for each patient. For fold f, every segment from patient f is used only for testing, and the model is fitted on the remaining 104 patients. Segment accuracy is calculated within each held-out patient. The 105 patient-specific accuracies are then averaged without weighting by segment count. This equally weighted LOSO mean is 71.29%. The corresponding distribution has a median of 78.17%, an SD of 24.22%, and a range of 0.00-96.00%.

The prediction unit is the 15-s ECG segment, while the resampling unit is the patient. Therefore, 71.29% is a patient-exclusive LOSO result but is distinct from a one-patient-one-label majority-vote accuracy. The code below reports both quantities separately to prevent this ambiguity.

S1.3. MK545Pat feature extractor

The function below implements the 58-index MK545Pat descriptor reported in the manuscript. Each 3,000-sample segment is scanned with 59-sample unit-stride blocks. The 59th sample is the pivot. The 58 comparisons are mapped to seven histograms, giving 2,304 features.

```
function histo = mk545pat58(signal)
% MK545PAT58  Deterministic MK545Pat descriptor used in the manuscript.
% Input : one 15-s ECG segment as a vector (3000 samples at 200 Hz)
% Output: 1 x 2304 histogram feature vector

signal = double(signal(:)');

pattern = [14 16 18 13 15 19 21 7 11 9 ...
           8 10 12 17 6 37 33 32 46 31 ...
           36 30 20 34 35 50 27 29 22 52 ...
           38 47 5 45 4 43 56 55 49 23 ...
           57 39 53 41 54 42 44 26 28 40 ...
           48 51 24 3 1 2 58 25];

assert(numel(pattern)==58 && isequal(sort(pattern),1:58), ...
    'MK545Pat pattern must be a permutation of 1:58.');

h1 = zeros(1,256); h2 = zeros(1,256); h3 = zeros(1,256);
h4 = zeros(1,256); h5 = zeros(1,256);
h6 = zeros(1,512); h7 = zeros(1,512);

for i = 1:(length(signal)-58)
    block = signal(i:i+58);
    pivot = block(59);
    bit = double(block(pattern) >= pivot);
```

```
    map1 = sum(bit(1:8)   .* 2.^(0:7));
    map2 = sum(bit(9:16)  .* 2.^(0:7));
    map3 = sum(bit(17:24) .* 2.^(0:7));
    map4 = sum(bit(25:32) .* 2.^(0:7));
    map5 = sum(bit(33:40) .* 2.^(0:7));
    map6 = sum(bit(41:49) .* 2.^(0:8));
    map7 = sum(bit(50:58) .* 2.^(0:8));

    h1(map1+1) = h1(map1+1) + 1;
    h2(map2+1) = h2(map2+1) + 1;
    h3(map3+1) = h3(map3+1) + 1;
    h4(map4+1) = h4(map4+1) + 1;
    h5(map5+1) = h5(map5+1) + 1;
    h6(map6+1) = h6(map6+1) + 1;
    h7(map7+1) = h7(map7+1) + 1;
end

histo = [h1 h2 h3 h4 h5 h6 h7];
end
```

S1.4. Patient-wise LOSO source code

The script below implements one held-out patient per fold. Feature standardization and NCA are fitted only on the training patients. The held-out patient is used only for prediction. The script calculates the equally weighted patient-specific LOSO accuracy, pooled held-out segment accuracy, and a separate patient majority-vote accuracy.

```
%% MK545Pat_patient_LOSO_FINAL.m
% Reference implementation of the patient-wise LOSO procedure used for the
% reported 71.29% cross-patient result.
%
% Required MAT-file variables:
%   segments  : N x 3000 matrix; one 15-s ECG segment per row
%   labels    : N x 1 labels, 1=AF, 2=PAF, 3=non-AF
%   subjectID : N x 1 patient identifier for each segment
%
% Data preparation used in the study:
%   - Native sampling rate: 200 Hz
%   - 15-s windows: 3000 samples
%   - Adjacent analysis windows: non-overlapping, stride = 3000 samples
%   - No additional signal-level filtering, denoising, amplitude
%     normalization, or resampling before MK545Pat
%
% The 59-sample unit-stride blocks are internal to MK545Pat and are not the
% stride used to create the 15-s analysis segments.

clear; clc; close all;

load('MK545Pat_LOSO_input.mat','segments','labels','subjectID');
segments = double(segments);
labels = double(labels(:));
subjectID = subjectID(:);

assert(size(segments,1)==numel(labels));
assert(numel(subjectID)==numel(labels));
assert(size(segments,2)==3000,'Each segment must contain 3000 samples.');
assert(all(ismember(unique(labels),1:3)),'Use 1=AF, 2=PAF, 3=non-AF.');

%% MK545Pat feature extraction
N = size(segments,1);
X = zeros(N,2304);
for n = 1:N
    X(n,:) = mk545pat58(segments(n,:));
end

%% Patient-wise LOSO
subjects = unique(subjectID,'stable');
nSub = numel(subjects);
yPred = nan(N,1);
score = nan(N,3);
foldID = nan(N,1);
```

```
selectedFeatures = cell(nSub,1);

for f = 1:nSub
    testMask = ismember(subjectID,subjects(f));
    trainMask = ~testMask;

    % The held-out patient must not appear anywhere in training.
    assert(isempty(intersect(unique(subjectID(trainMask)), ...
                             unique(subjectID(testMask)))), ...
           'Patient leakage detected in fold %d.',f);

    % Feature standardization is fitted on training patients only.
    mu = mean(X(trainMask,:),1);
    sigma = std(X(trainMask,:),0,1);
    sigma(sigma==0) = 1;
    Xtr = (X(trainMask,:)-mu)./sigma;
    Xte = (X(testMask,:)-mu)./sigma;

    % NCA is fitted on training patients only.
    nca = fscnca(Xtr,labels(trainMask),'Solver','lbfgs','Verbose',0);
    [~,ranked] = sort(nca.FeatureWeights,'descend');
    sel = ranked(1:min(1000,numel(ranked)));
    selectedFeatures{f} = sel;

    % Degree-3 polynomial SVM for the three-class task.
    t = templateSVM('KernelFunction','polynomial', ...
                    'PolynomialOrder',3, ...
                    'Standardize',false);
    model = fitcecoc(Xtr(:,sel),labels(trainMask), ...
                     'Learners',t,'Coding','onevsone');

    [yPred(testMask),sc] = predict(model,Xte(:,sel));
    if size(sc,2)==3
        score(testMask,:) = sc;
    end
    foldID(testMask) = f;
end

%% LOSO summaries
order = [1 2 3];
CM_segment = confusionmat(labels,yPred,'Order',order);
segmentPooledAccuracy = sum(diag(CM_segment))/sum(CM_segment(:));

truePatient = nan(nSub,1);
predPatient = nan(nSub,1);
nSegments = zeros(nSub,1);
patientSegmentAccuracy = nan(nSub,1);

for f = 1:nSub
    m = ismember(subjectID,subjects(f));
    nSegments(f) = sum(m);
    truePatient(f) = mode(labels(m));
    predPatient(f) = mode(yPred(m));
    patientSegmentAccuracy(f) = mean(labels(m)==yPred(m));
end

% This is the aggregation rule used for the reported 71.29% LOSO result:
% every held-out patient contributes one equally weighted segment accuracy.
patientWeightedLOSOAccuracy = mean(patientSegmentAccuracy);

% This is a different endpoint and must not be confused with 71.29%.
CM_patientVote = confusionmat(truePatient,predPatient,'Order',order);
patientMajorityVoteAccuracy = sum(diag(CM_patientVote))/sum(CM_patientVote(:));

%% Save auditable outputs
Tsegment = table(string(subjectID),foldID,labels,yPred, ...
    score(:,1),score(:,2),score(:,3), ...
    'VariableNames',{'Subject','Fold','TrueLabel','PredictedLabel', ...
                     'Score_AF','Score_PAF','Score_nonAF'});
writetable(Tsegment,'MK545Pat_LOSO_segment_predictions.csv');

Tpatient = table(string(subjects),nSegments,truePatient,predPatient, ...
```

```
    100*patientSegmentAccuracy, ...
    'VariableNames',{'Subject','NSegments','TrueLabel','MajorityVoteLabel', ...
                     'HeldOutSegmentAccuracy_percent'});
writetable(Tpatient,'MK545Pat_LOSO_patient_summary.csv');

writematrix(CM_segment,'MK545Pat_LOSO_segment_confusion.csv');
writematrix(CM_patientVote,'MK545Pat_LOSO_patient_vote_confusion.csv');

fprintf('Pooled held-out segment accuracy       : %.2f%%\n',100*segmentPooledAccuracy);
fprintf('Patient-weighted LOSO accuracy         : %.2f%%\n',100*patientWeightedLOSOAccuracy);
fprintf('Patient majority-vote accuracy         : %.2f%%\n',100*patientMajorityVoteAccuracy);
fprintf('Median patient-specific accuracy       : %.2f%%\n',100*median(patientSegmentAccuracy));
fprintf('SD of patient-specific accuracies      : %.2f%%\n',100*std(patientSegmentAccuracy));
fprintf('Range of patient-specific accuracies   : %.2f-%.2f%%\n', ...
        100*min(patientSegmentAccuracy),100*max(patientSegmentAccuracy));
```
